# Unilateral Stereotactic Radiofrequency Lesioning as a Surgical Treatment Option for Meige Syndrome

**DOI:** 10.7759/cureus.67064

**Published:** 2024-08-17

**Authors:** Yoji Kuramoto, Takaomi Taira, Shoichiro Tsuji, Shinichi Yoshimura

**Affiliations:** 1 Neurosurgery, Hyogo Medical University, Nishinomiya, JPN; 2 Functional Neurosurgery, Kumagaya General Hospital, Kumagaya, JPN

**Keywords:** thermal radiofrequency ablation, lesioning in dystonia, stereotactic and functional neurosurgery, functional neurological disorder, facial dystonia, meige syndrome

## Abstract

Background

Meige syndrome is a segmental dystonia affecting the head and neck, with bilateral blepharospasm as the primary symptom. First-line treatment typically involves Botox injections. For cases resistant to this treatment, bilateral deep brain stimulation of the globus pallidus internus (GPi) is considered. This study explores the efficacy of unilateral radiofrequency (RF) lesioning as an alternative surgical treatment for Meige syndrome.

Methods

We investigated six cases of medically refractory Meige syndrome treated with unilateral RF lesioning between October 2022 and August 2023. The procedures utilized the Leksell Stereotactic System (Elekta, Stockholm, Sweden) and the StealthStation S8 system (Medtronic, Dublin, Ireland). Target coordinates were initially set at 8-9 mm lateral and 1-2 mm inferior to the mid-commissure point (MCP) for the pallidothalamic tract (PTT), and 20 mm lateral, 2 mm anterior, and 3.0-4.5 mm inferior to the MCP for GPi, with fine adjustments based on MRI findings.

Results

The mean age of patients was 53. 3 ±16.5 years. Five patients underwent PTT RF lesioning, while one received GPi RF lesioning (pallidotomy). No surgical complications were reported. The Burke-Fahn-Marsden Dystonia Rating Scale scores were 32.9 ± 19.4 preoperatively and 17.7 ± 13.9 three months postoperatively, reflecting an average improvement of 42.7%. The Jankovic Rating Scale scores were 7.17 ± 0.76 preoperatively, 2.33 ± 2.34 the day after surgery (average improvement of 67%), and 3.50 ± 1.64 three months postoperatively (average improvement of 51%). Bilateral facial symptoms improved in four patients (67%).

Conclusion

Unilateral RF lesioning for Meige syndrome demonstrated the potential to improve bilateral symptoms and may be considered a viable treatment option for patients with refractory cases.

## Introduction

Meige syndrome is a segmental dystonia affecting the head and neck, characterized by blepharospasm and temporomandibular joint dystonia. This condition often involves complex muscle movements of the lower face, mouth, jaw, tongue, pharynx, and neck. Blepharospasm is frequently the earliest clinical manifestation. Botox injections have become the first-line treatment [1], while deep brain stimulation (DBS) is considered for cases resistant to Botox and other medical treatments [2,3]. However, DBS can be associated with infections and equipment issues [4], and battery replacement surgeries are also required.

Radiofrequency (RF) lesioning, initially performed by Wycis and Spiegel in the 1950s for various involuntary movement disorders, has declined in use with the advent of DBS. While Meige syndrome typically presents bilaterally, necessitating bilateral treatment and potentially favoring DBS, our series has shown that unilateral RF lesioning can lead to bilateral symptomatic improvement in some cases. This procedure, performed under local anesthesia and lasting about 30 minutes, offers an alternative to DBS. Nonetheless, unilateral RF lesioning may result in limited symptom improvement in some patients, and predicting the surgical outcome preoperatively can be challenging. It is important to thoroughly discuss these considerations with patients before proceeding with unilateral RF lesioning.

## Materials and methods

We conducted a retrospective review of six consecutive cases of Meige syndrome in which unilateral RF lesioning was performed between October 2022 and August 2023. At our institution, we discuss both RF lesioning and DBS with patients whose dystonia, including Meige syndrome, is refractory to medical therapy and local Botox injections, helping them choose the most suitable treatment. However, RF lesioning is available at only a few centers in our region, leading to referrals for this procedure.

Given the potential complications and risks of simultaneous bilateral RF lesioning, we opted for unilateral RF lesioning as an initial intervention. We then consulted with the patients to determine whether contralateral RF lesioning should be considered after a six-month period. This study focuses on evaluating the outcomes of the initial unilateral RF lesion.

To determine the surgical side, the more symptomatic side of the brain, typically the contralateral side, was initially chosen for surgery. If symptoms were more pronounced on one side, that side was selected for intervention. In cases where symptoms were bilateral or similar, the nondominant hemisphere, usually the right side, was preferred for the initial surgery. This approach is based on the understanding that the dominant hemisphere may be more susceptible to severe side effects from an intracerebral hemorrhage, making it preferable to operate on the nondominant hemisphere when possible.

Regarding the lesion site, the standard approach was to target the pallidothalamic tract (PPT) in the nondominant hemisphere and the globus pallidus internus (GPi) in the dominant hemisphere. PPT lesioning has the drawback of potentially causing transient muscle hypotonia, which can significantly impact daily life and employment, particularly if it affects the dominant arm. Therefore, the nondominant hemisphere, and by extension, the nondominant arm, was selected to minimize these risks.

Stereotactic surgery was performed using the Leksell Stereotactic System (Elekta, Stockholm, Sweden) in all cases. Planning was done with the StealthStation S8 system (Medtronic, Dublin, Ireland), targeting 8-9 mm lateral and 1-2 mm inferior to the mid-commissure point (MCP) for the PPT and 20 mm lateral, 2 mm anterior, and 3.0-4.5 mm inferior to the MCP for the GPi. These targets were fine-tuned using preoperative MRI, which included 1 mm-thick T1-weighted and T2-weighted images.

On the day of surgery, a CT scan was performed after fitting the Leksell frame, and the CT was fused with the MRI for accurate targeting. A monopolar lesioning needle with a 1 mm tip width (No. 1017044, Elekta) was used, capable of coagulating up to 4 mm from the tip. The needle was navigated to the predetermined target using the Leksell Stereotactic System, inserted slowly, and its electrical resistance was monitored to ensure it followed the planned trajectory. Test stimulation and coagulation were conducted using a Leksell Neurogenerator (Elekta).

The severity of clinical symptoms was evaluated preoperatively and three months postoperatively using the Burke-Fahn-Marsden Dystonia Rating Scale (BFMDRS). Blepharospasm severity was assessed with the Jankovic Rating Scale (JRS) preoperatively, on the day of surgery, and three months postoperatively. Perioperative changes and complications were also recorded. Statistical analysis was performed using the Wilcoxon test to compare preoperative values with postoperative or three-month values, with a significance threshold set at P < 0.05.

## Results

The cohort consisted of three male and three female patients, with an average age of 53.3 ± 16.5 years. Among the six patients, five underwent PTT lesioning, while one underwent GPi lesioning (pallidotomy). Basic characteristics are detailed in Table 1.

**Table 1:** Basic characteristics of all patients BFMDRS: Burke-Fahn-Marsden Dystonia Rating Scale; GPi: globus pallidus internus; JRS: Jankovic Rating Scale; PTT: pallidothalamic tract

Patient no.	Age	Gender	Pre-BFMDRS	Pre-JRS	Operated side	Target
1	60s	Male	48	8	Right	PTT
2	20s	Female	34	7	Right	PTT
3	40s	Female	14	6	Left	PTT
4	70s	Female	28	7	Right	PTT
5	50s	Male	62	7	Left	GPi
6	50s	Male	12	8	Right	PTT

**Figure 1:**
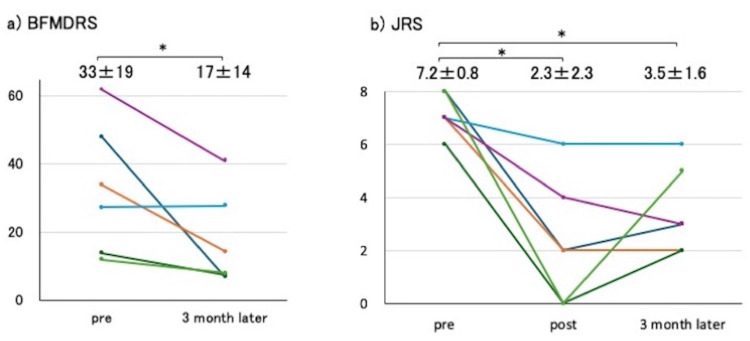
Improvement in clinical scores following unilateral lesioning (a) The BFMDRS score was 32.9 ± 19.4 preoperatively and 17.7 ± 13.9 three months postoperatively, showing an average improvement of 42.7%. (b) The JRS score was 7.17 ± 0.76 preoperatively and 2.33 ± 2.34 immediately postoperatively, reflecting an average improvement of 67%. Three months postoperatively, the JRS score was 3.50 ± 1.64, indicating a further average improvement of 51%. BFMDRS: Burke-Fahn-Marsden Dystonia Rating Scale; JRS: Jankovic Rating Scale

The preoperative BFMDRS score was 32.9 ± 19.4, which improved to 17.7 ± 13.9 three months postoperatively, representing a mean improvement of 42.7% (Figure 1a).

The preoperative JRS was 7.17 ± 0.76, improving to 2.33 ± 2.34 the day after surgery, reflecting a mean improvement of 67%. Three months postoperatively, the JRS was 3.50 ± 1.64, indicating a mean improvement of 51% (Figure 1b). Despite performing unilateral surgery, four patients (67%) experienced an improvement in bilateral facial symptoms. No surgical complications were observed during the perioperative period.

A representative case involves a left-handed female in her 40s who had been managing bilateral facial spasms with Botox treatment for over 10 years. Approximately two years ago, she developed dysphagia following Botox injections, which complicated ongoing treatment. Subsequently, her symptoms worsened, with increased neck forward bending and bilateral facial spasms during walking. Additionally, she experienced a change in voice, leading to a referral for surgical treatment.

**Figure 2:**
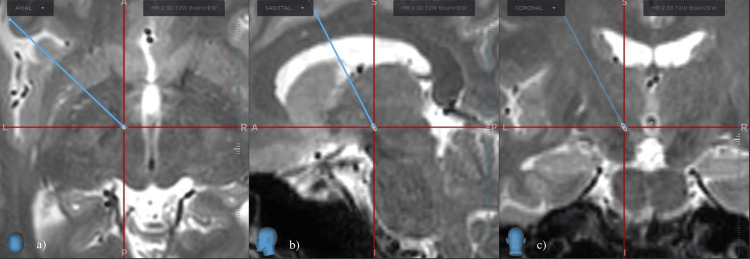
Preoperative MRI Preoperative MRI T2-weighted imaging with (a) axial, (b) sagittal, and (c) coronal views. The blue line denotes the planned trajectory, while the intersection of the red lines represents the planned target.

The primary symptoms included bilateral eyelid spasm, neck forward flexion, and altered voice, with a BFMDRS score of 16 and a JRS score of 6. Given the patient’s left-handedness and symmetric symptoms, we selected the left PTT for intervention. Tentative target coordinates were 8.5 mm lateral and 1-2 mm inferior to the MCP (Figure 2).

A unilateral posterior thalamic thalamotomy was performed. The day after surgery, bilateral blepharospasm and cervical anterior flexion resolved. Voice changes showed partial improvement, with increased time for the voice to become clear without the need for sensory tricks. Three months later, the symptoms were assessed with a BFMDRS score of 7.5 and a JRS score of 2 (Video 1).

**Video 1:** Representative patient movie Preoperatively, the patient exhibited blepharospasm, cervical anterior flexion, and voice stammering. During surgery, a lesioning needle was inserted into the medial left PTT. The lesioning was performed after observing muscle tonus improvement with test stimulation and noting that speaking became easier. The procedure was extended to include the lateral left PTT, and the operation was completed as planned on the MRI. Postoperatively, the patient demonstrated symptom improvement, with the positive effects lasting for six months following the surgery.

**Figure 3:**
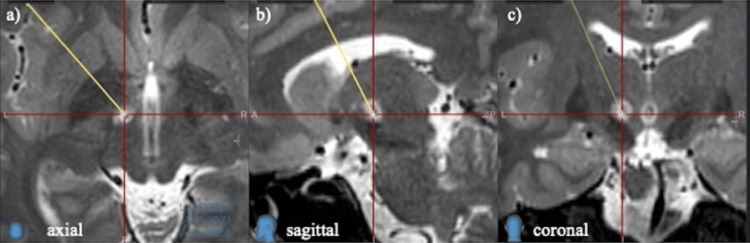
Postoperative MRI Postoperative MRI T2-weighted images in (a) axial, (b) sagittal, and (c) coronal planes are shown. The yellow line represents the planned trajectory, and the intersection of the red lines indicates the planned target.

The postoperative MRI confirmed that the lesioning site was correctly targeted (Figure 3).

## Discussion

Meige syndrome is a type of segmental dystonia characterized by blepharospasm and involuntary movements in adjacent areas. The condition was first described by Wood in 1887, who reported blepharospasm and other facial dystonias [5]. In 1910, Meige documented 10 cases of involuntary eyelid closure and, in one case, involuntary jaw muscle contraction [6]. In 1972, Paulson identified eyelid and facial dyskinesia as Meige syndrome [7]. For treatment-resistant Meige syndrome, therapeutic surgery is an option, with DBS often employed [2,3]. However, DBS carries risks, including infections, skin damage from exposure to generators and leads, and equipment malfunctions [4]. Battery replacement surgeries are also necessary, and the high cost of DBS equipment poses economic challenges. DBS targets include both the subthalamic nuclei and the GPi, with effectiveness considered equivalent [8], although the GPi is more commonly used. Unilateral pallidotomy has also shown improvement in Meige syndrome [9]. However, since Meige syndrome frequently presents bilaterally, treating only one side may not provide sufficient benefit.

Bilateral pallidotomy has shown similar effectiveness to bilateral GPi-DBS but is associated with a higher incidence of complications such as dysphagia, dysarthria, and parkinsonism compared to unilateral pallidotomy [10,11]. Consequently, DBS is often preferred for bilateral surgery. To mitigate the risks associated with bilateral pallidotomy, staged lesion resection has been proposed [12]. This approach reduces complications by avoiding simultaneous bilateral surgery at the same site [13,14].

In this report, a two-stage surgical approach was employed: lesioning of one side of the GPi in the first stage and PTT lesioning on the opposite side in the second stage, with a six-month interval between surgeries. This method was as effective as bilateral pallidotomy, with no severe complications affecting daily life [14]. Based on this experience, an alternative approach involves performing staged lesioning with different targets on each side (e.g., PTT first, GPi second, or vice versa) as an alternative to DBS for treating head and neck dystonia.

In our series, symptomatic improvement has been observed even with unilateral lesioning alone. While clinical and laboratory tests cannot predict which side of the brain is most involved with the symptoms, it is likely that initial unilateral lesioning targets the more affected side. However, the effectiveness of this approach varies among patients, and in some cases, unilateral lesioning alone does not result in symptom improvement. Therefore, predicting the preoperative effects of surgery is challenging. It is crucial to thoroughly explain this variability to patients considering unilateral lesions of the PTT or GPi.

The PTT, or Forel H field, is a pathway connecting the GPi to the thalamus and was initially used as a target for epilepsy [15]. Recently, it has also proven effective in treating dystonia [16]. Unilateral PTT lesioning is considered a promising initial approach due to its lower risk of lacunar infarction compared to GPi [17,18] and fewer complications than bilateral pallidotomy. It also allows for the possibility of future contralateral GPi lesions. Patients should be advised about the risk of falls following unilateral PTT lesioning, as it can result in transient mild muscle weakness more frequently than pallidotomy [16,19].

A limitation of this study is the small sample size, which may result in infrequent side effects going undetected. If future imaging studies can identify a more effective surgical target, unilateral lesioning could become a more viable option for treating dystonia with bilateral symptoms, including Meige syndrome [20].

## Conclusions

Unilateral RF lesioning is considered a viable surgical treatment option for Meige syndrome, especially in cases where general anesthesia is challenging, patients are at high risk for bilateral DBS, or those who are averse to having implanted devices. However, given the small number of cases to date, further research with a larger patient population is needed to confirm its efficacy and safety.
